# Variation in Classification and Postoperative Management of Complex Appendicitis: A European Survey

**DOI:** 10.1007/s00268-018-4806-4

**Published:** 2018-09-25

**Authors:** Elisabeth M. L. de Wijkerslooth, Anne Loes van den Boom, Bas P. L. Wijnhoven

**Affiliations:** Department of Surgery, Suite Na-2117, Erasmus MC – University Medical Centre, PO Box 2040, 3000 CA Rotterdam, The Netherlands

## Abstract

**Background:**

Data on common practice in the management of patients with complex appendicitis are scarce, especially for the adult population. Variation in the definition of complex appendicitis, indications for and the type of prolonged antibiotic prophylaxis have not been well studied yet. The aim of this study was to document current practice of the classification and postoperative management of complex appendicitis on an international level.

**Methods:**

An online survey was dispersed among practicing surgeons and surgical residents. Survey questions pertained to the definition of a complex appendicitis, indications for antibiotic prophylaxis after appendectomy, the duration, route of administration and antibiotic agents used.

**Results:**

A total of 137 survey responses were eligible for analysis. Most respondents were from Northern or Western Europe and were specialized in gastrointestinal surgery. Opinion varied substantially regarding the management of appendicitis, in particular for phlegmonous appendicitis with localized pus, gangrenous appendicitis and iatrogenic rupture of appendicitis. The most common duration of postoperative antibiotics was evenly spread over <3, 3, 5 and 7 days. Whereas most respondents indicated a combined intravenous and oral route of administration was common practice, 28% answered a completely intravenous route of administration was standard practice.

**Conclusion:**

Current practice patterns in the classification and postoperative management of complex appendicitis are highly variable.

**Electronic supplementary material:**

The online version of this article (10.1007/s00268-018-4806-4) contains supplementary material, which is available to authorized users.

## Introduction

Acute appendicitis is a highly prevalent surgical emergency in both children and adults [1–4]. Yet, the optimum management of this disease remains a subject of controversy. The non-operative management is increasingly being studied, but emergency appendectomy remains the cornerstone of treatment in most hospitals [5–7]. If the surgeon classifies the type of appendicitis as complex, antibiotic prophylaxis should be continued after surgery [8–11]. This aims to prevent infectious complications, including recurrent intra-abdominal infections. The available guidelines recommend to extend prophylaxis for 3–7 postoperative days [8–13]. The alarming emergence of antimicrobial resistance worldwide warrants optimization of antibiotic use, as presented as a key focus by the WHO [14]. Therefore, it is key to carefully select patients that benefit from prolonged prophylaxis and to define the most optimal regimen.

A survey among Dutch surgeons demonstrated that a clear standard of care is missing both in patient selection and in determining the length of treatment [15]. The definition of complex appendicitis used in studies varies. Apart from its common component: perforated appendicitis, it may or may not also include unperforated gangrenous appendicitis, appendicitis in the presence of a faecolith and/or appendicitis in the presence of pus, or purulent peritonitis, or abscess [16–20]. Postoperative antibiotic use is left to the discretion of the surgeon. Five days of antibiotics, switched from an intravenous to oral route as early as 48 h after surgery, is common use in many centers in the Netherlands [15, 16]. Another strategy, which is gaining ground, consists of 3 days of intravenous antibiotics only [15, 16, 21]. Intravenous regimens most used are cefuroxime or ceftriaxone in combination with metronidazole [22]. Amoxicillin–clavulanate is often chosen as oral antibiotic. Little is reported in the literature regarding the common practice of prolonged prophylaxis after appendectomy in other countries. Some studies have reported variability in care for patients with complex appendicitis [23–29]. Most studies included only pediatric patients, and few focused on the postoperative management of appendicitis. In pursuit of the optimum antibiotic regimen for complex appendicitis, a variety of treatment protocols have been reported [16, 21, 30–33]. Limiting antibiotic use to 5 days at most is widely accepted, but no specific duration of postoperative antibiotic use has proven most optimal. Previous research has shown that standardization of practice can be beneficial in terms of clinical outcomes after appendectomy (i.e., postoperative abscess formation and length of hospital stay) [29, 34]. Identifying variation in practice may therefore reveal opportunities for quality improvement.

The aim of this study was to determine the variation in the classification and postoperative management of complex appendicitis on an international level.

## Materials and methods

The present study was a cross-sectional, international, anonymous online survey among surgeons and surgical residents, which took place from June until September 2017. Several surgical associations and research collaboratives (European Digestive Surgery; East Midlands Surgical Academic Network; GlobalSurg; National Research Collaborative (UK/Ireland); Scottish Surgical Research Group; South Yorkshire Surgical Research Group;, West Midlands Research Collaborative) kindly dispersed the survey among their members. Through email, surgeons and residents were invited to participate by clicking a link to enter the online survey module. Three to four weeks after the first email, a second reminder was sent out. Participation was voluntary. Due to widespread dispersion of the survey through association newsletters and personal forwarding response rate could not be assessed.

The survey consisted of thirteen questions in total. Data on the respondents’ backgrounds were collected in the first five questions. Next, respondents were to answer two questions *based on their personal professional opinion*: concerning the definition of a complex appendicitis and indications for prolonged antibiotic prophylaxis after appendectomy. Lastly, respondents were to answer five questions *based on common practice at their hospital*: these were questions regarding the duration, route of administration and antibiotic agents used as prolonged prophylaxis after appendectomy. All survey questions were multiple-choice questions. Only 4 questions allowed for a free-text answer if answer option ‘Other’ was ticked. The full survey question list can be found in supplementary file S1.

### Statistics

All survey data were analyzed by means of simple descriptive statistics using Excel^®^ 2010 (Microsoft, Redmond, Washington, USA) and SPSS version 21 (IBM, Armonk, New York, USA). Included in the analysis are results from all European respondents that completed at least the survey items on the definition of a complex appendicitis.

## Results

A total of 150 European respondents submitted their surveys within the 2-month time frame. Ten responses were excluded from the analysis due to insufficient completion. Another three were excluded, as the respondents were not surgeons or surgical residents. The remaining 137 surveys were analyzed. The respondents were employed in 82 different hospitals in 19 countries. Background characteristics of the respondents are shown in Table 1. Eighty-four percent of them performed appendectomy at least monthly.

**Table 1:** Study participants (n = 137)

	*n* (%)
Region^a^	
Northern Europe	76 (55)
Western Europe	48 (35)
Other	13 (10)
Profession	
Surgeon	84 (61)
Senior resident (4th–6th year)	28 (20)
Junior resident (1st–3rd year)	25 (18)
Field of specialization^b^	
Gastrointestinal/oncological surgery	110 (80)
Trauma surgery	12 (9)
Vascular surgery	6 (4)
General surgery	6 (4)
Other^c^	7 (5)
No differentiation (yet)	16 (12)
Type of hospital	
Academic or university hospital	83 (61)
General hospital	30 (22)
Teaching hospital	22 (16)
Other^d^	2 (1)
Performs appendectomy	
Rarely (<1 per month)	22 (16)
Sometimes (1–2 per month)	34 (25)
Often (>2 per month)	81 (59)

^a^Number of respondents per country is available in Supplementary Table S1

^b^More than one answer was allowed

^c^Other specializations included: 4 × emergency surgery, 1 × hand surgery, 1 × orthopedics and 1 × pediatric surgery

^d^Other answer included: 1 × private clinic, 1 × general pediatric teaching hospital

### Definition of complex appendicitis and indications for prolonged prophylaxis (Table 2)

**Fig. 1:**
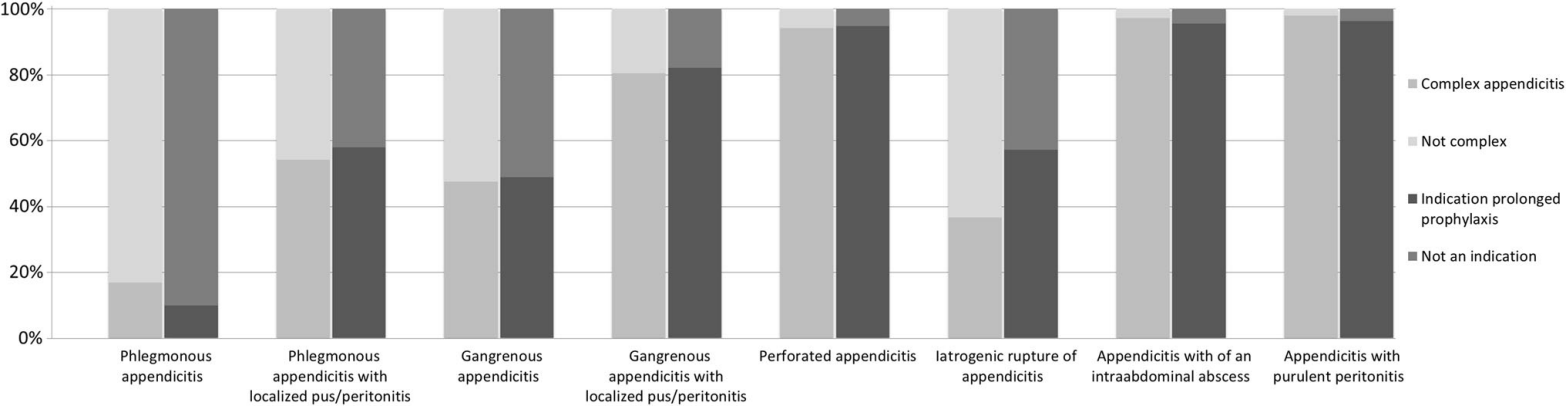
Definition of a complex appendicitis and indications for prolonged antibiotic prophylaxis

Eighty-eight percent of respondents was familiar with the classification of appendicitis into simple and complex appendicitis; fifty percent indicated they most often used the classification in practice. For the 8 types of appendicitis used in this survey, the proportion of surgeons that considered it a complex appendicitis type and the proportion that considered it an indication for prolonged prophylaxis are shown in Table 2. Disagreement among the respondents, especially regarding phlegmonous appendicitis with localized pus/peritonitis, gangrenous appendicitis and iatrogenic rupture of appendicitis, is further illustrated in Fig. 1.

**Table 2:** Respondents’ answers on the definition of complex appendicitis and indication for postoperative antibiotic use, *n* (%)

	All *n *= 137	Northern Eur. *n *= 76	Western Eur. *n *= 48	Other *n *= 13
Do you consider the following types of appendicitis complex? *Answer ‘yes’*				
Phlegmonous appendicitis	23 (17)	10 (13)	10 (21)	3 (23)
Phlegmonous appendicitis with localized pus/peritonitis	74 (54)	34 (45)	32 (67)	8 (62)
Gangrenous appendicitis	65 (47)	31 (41)	26 (54)	8 (62)
Gangrenous appendicitis with localized pus/peritonitis	110 (80)	57 (75)	41 (85)	12 (92)
Perforated appendicitis	129 (94)	73 (96)	44 (92)	12 (92)
Iatrogenic rupture of appendicitis	50 (36)	33 (43)	12 (25)	5 (38)
Appendicitis with of an intra-abdominal abscess	133 (97)	74 (97)	47 (98)	12 (92)
Appendicitis with purulent peritonitis	134 (98)	76 (100)	46 (96)	12 (92)

	All *n *= 133	Northern Eur. *n *= 73	Western Eur. *n *= 47	Other *n *= 13
Do the following patients need postoperative antibiotic treatment? *Answer ‘yes’*				
Patient with phlegmonous appendicitis	13 (10)	2 (3)	9 (19)	2 (15)
Patient with phlegmonous appendicitis with localized pus/peritonitis	77 (58)	37 (51)	30 (64)	10 (77)
Patient with gangrenous appendicitis	65 (49)	31 (42)	23 (49)	11 (85)
Patient with gangrenous appendicitis with localized pus/peritonitis	109 (82)	59 (81)	37 (79)	13 (100)
Patient with perforated appendicitis	126 (95)	71 (97)	43 (91)	12 (92)
Patient with iatrogenic rupture of appendicitis	76 (57)	38 (52)	28 (60)	10 (77)
Patient with appendicitis with of an intra-abdominal abscess	127 (95)	69 (95)	46 (98)	12 (92)
Patient with appendicitis with purulent peritonitis	128 (96)	70 (96)	46 (98)	12 (92)

### Duration, route of administration and antibiotic agents

**Fig. 2:**
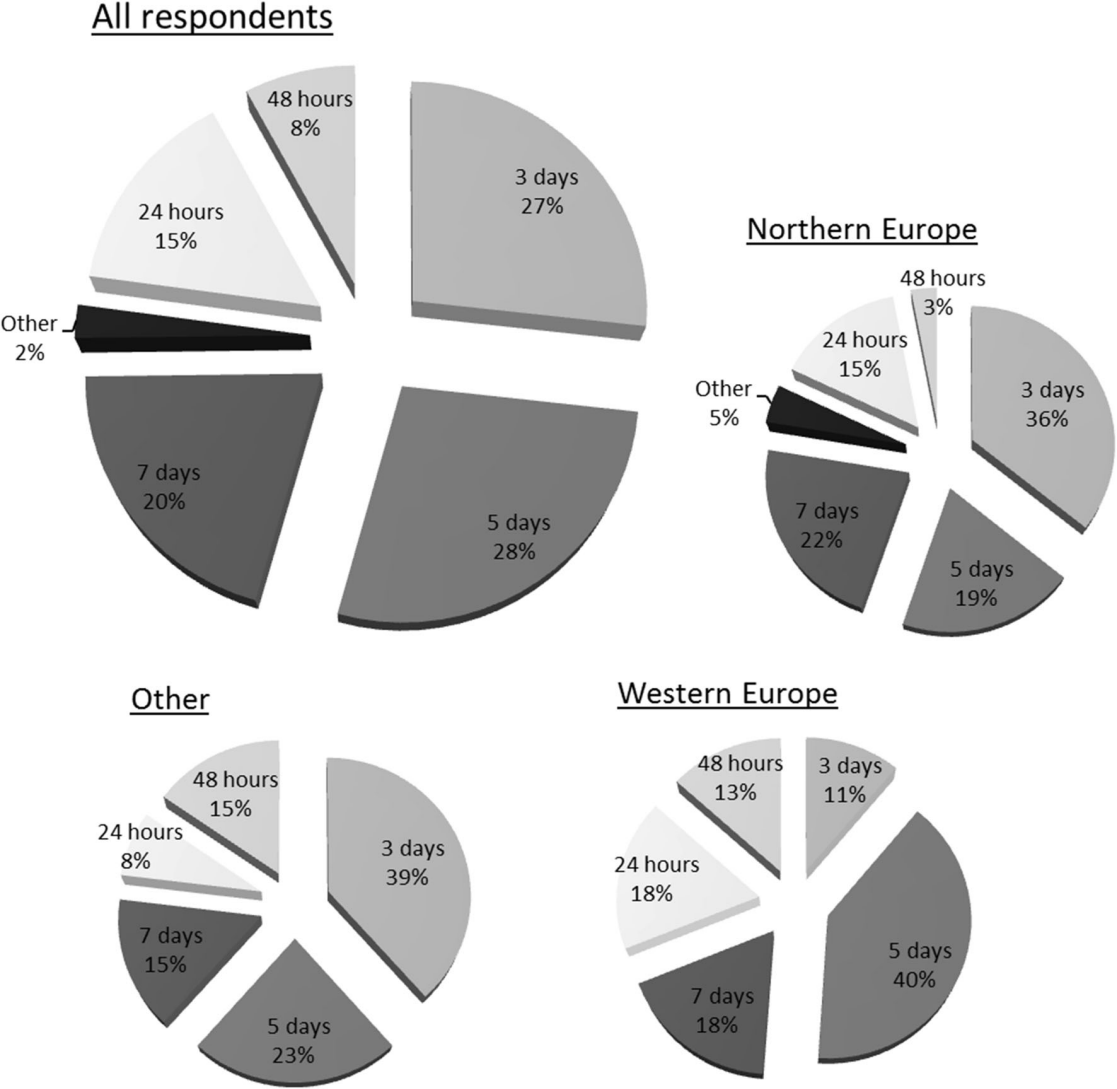
Most common duration of prolonged antibiotic prophylaxis for complex appendicitis. *Northern Europe* Denmark, Finland, Lithuania, Norway and Sweden. *Western Europe* Belgium, Germany, Ireland, Switzerland and the UK. *Other* Bulgaria, Croatia, Greece, Italy, Poland, Romania, Spain, Turkey

Table 3 shows the variation in treatment duration and route of administration, according to the respondents’ answers on policy at their hospital. Forty-five percent of respondents answered that the minimum duration of prolonged prophylaxis at their hospital was 24 or 48 h. Subsequently, 23 percent indicated that this was the most common duration (Fig. 2). Most respondents that indicated 24 h as minimum were from the UK (49%) or Finland (24%). The majority answered that a combined intravenous and oral course was most prescribed at their hospital (Table 3). The most popular intravenous antibiotic regimens were cefuroxime in combination with metronidazole (27%), amoxicillin/clavulanate (22%) and piperacillin in combination with tazobactam (12%). And the most preferred oral agents were amoxicillin/clavulanate (37%), ciprofloxacin in combination with metronidazole (24%) and cephalexin in combination with metronidazole (11%).

**Table 3:** Respondents’ answers on duration and administration of postoperative antibiotic use for complex appendicitis at their hospital, n (%)

	All *n *= 127	Northern Eur. *n *= 68	Western Eur. *n *= 46	Other *n *= 13	Denmark *n *= 16	Finland *n *= 19	Ireland *n *= 10	Lithuania *n *= 12	Norway *n *= 13	UK *n *= 29
Minimum duration										
24 h	45 (35)	15 (22)	27 (59)	3 (23)	1 (6)	11 (58)	5 (50)	1 (8)	1 (8)	22 (76)
48 h	13 (10)	3 (4)	5 (11)	5 (38)	0	2 (11)	1 (10)	1 (8)	0	3 (10)
3 days	46 (36)	37 (54)	5 (11)	4 (31)	15 (94)	3 (16)	1 (10)	5 (42)	11 (85)	2 (7)
5 days	18 (14)	10 (15)	7 (15)	1 (8)	0	3 (16)	2 (20)	4 (33)	1 (8)	1 (3)
7 days	5 (4)	3 (4)	2 (4)	0	0	0	1 (10)	1 (8)	0	1 (3)

	All *n *= 127^a^	Northern Eur. *n *= 67^a^	Western Eur. *n *= 45	Other *n *= 13	Denmark *n *= 16	Finland *n *= 19	Ireland *n *= 10	Lithuania *n *= 12^b^	Norway *n *= 13^b^	UK *n *= 28
Most common duration										
24 h	19 (15)	10 (12)	8 (18)	1 (8)	1 (6)	4 (20)	0	3 (25)	0	8 (29)
48 h	10 (8)	2 (2)	6 (13)	2 (15)	0	1 (5)	1 (10)	1 (8)	0	5 (18)
3 days	34 (27)	24 (39)	5 (11)	5 (38)	15 (94)	1 (5)	1 (10)	2 (17)	4 (31)	4 (14)
5 days	35 (28)	13 (19)	18 (40)	3 (23)	0	5 (26)	6 (60)	3 (25)	4 (31)	8 (29)
7 days	26 (20)	15 (25)	8 (18)	2 (15)	0	8 (40)	2 (20)	2 (17)	4 (31)	3 (11)

	All *n *= 130	Northern Eur. *n *= 70	Western Eur. *n *= 47	Other *n *= 13	Denmark *n *= 17	Finland *n *= 19	Ireland *n *= 10	Lithuania *n *= 12	Norway *n *= 13	UK *n *= 30
Common administration										
Intravenous (IV)	36 (28)	23 (30)	8 (17)	5 (38)	8 (47)	3 (16)	3 (30)	5 (42)	4 (31)	5 (17)
Combined (IV/PO)	93 (72)	46 (61)	39 (81)	8 (62)	8 (47)	16 (84)	7 (70)	7 (58)	9 (69)	25 (83)
Oral (PO)	1 (1)	1 (1)	0	0	1 (6)	0	0	0	0	0

Results shown for all respondents, per region and per country with at least 10 respondents that completed the relevant survey items

^a^Three other responses: 2 × 4 days and 1 × 10 days

^b^One other response: 4 days

## Discussion

The present study was designed to provide an overview of current practice in the postoperative management of complex appendicitis. There was a considerable variation in the definition of a complex appendicitis, indications for prolonged antibiotic prophylaxis after appendectomy and the antibiotic regimens used. Such variation in practice may have an effect on clinical outcomes, and standardization may impact the appropriate use of antibiotics worldwide given the rising antimicrobial resistance.

The vast majority of surgeons in this survey agreed that appendicitis with perforation, intra-abdominal abscess or purulent peritonitis can be defined as complex appendicitis for which prolonged antibiotic prophylaxis is indicated. Most respondents (80%) also classified a gangrenous appendicitis with localized pus as complex appendicitis. Opinion was divided regarding a gangrenous appendicitis without localized pus: only about half considered this type a complex appendicitis. In their guideline on intra-abdominal infections, the Surgical Infection Society and Infectious Diseases Association of America recommend to restrict antibiotic prophylaxis to 24 h after appendectomy for gangrenous unperforated appendicitis [10]. Nevertheless, as confirmed in this survey, some clinicians feel that a gangrenous appendicitis increases the patient’s risk of an infectious complication and there is some evidence that supports this [35]. Responses were ambiguous for phlegmonous appendicitis with localized pus as well. It appears that the presence of (localized) pus in the abdomen could be a decisive factor for some surgeons to classify appendicitis as complex. However, none of the available guidelines take into account the presence of pus in the decision of prescribing postoperative antibiotics (nor do they mention abscess or purulent peritonitis) [9–11]. Strikingly, 36% of respondents felt that a iatrogenic rupture of appendicitis fell within the definition of a complex appendicitis, yet 57% indicated that postoperative antibiotics were needed. Such variation in opinion among surgeons may originate from a lack of consensus in the literature, especially literature on adult patients [12, 13, 36, 37]. These results imply that depending on the type of appendicitis, a patient might be treated completely different by one surgeon compared to another. The present analyses showed that this standard care differs substantially for several types of appendicitis, both between and within countries.

The most common duration of prolonged prophylaxis for complex appendicitis was almost evenly spread over less than 3 days, 3, 5 and 7 days. About half the respondents answered that prophylaxis was most often extended *beyond* 3 postoperative days. A large prospective cohort study demonstrated that 3 and 5 days of postoperative antibiotics result in similar rates of infectious complications [21]. Thus, substantial overtreatment may exist. Another sign of potential overuse of antibiotics is that there was quite a large difference between the minimum durations in hospital protocols and the most commonly practiced durations. Randomized studies will have to confirm whether a reduced course is indeed safe and effective. In this survey, responses from Denmark were unambiguous: all but one indicated that 3 days of postoperative antibiotics was the minimum as well as the most common duration. This duration has become the standard in Denmark [38]. For the remainder, responses on duration varied greatly within and between geographical regions. Again, this implies considerable variation in care. For one individual patient, this may affect their length of stay in the hospital and perhaps their risk of an infectious complication. Moreover, on a national or international level, a reduced or prolonged antibiotic course may have a significant impact on antibiotic use, antimicrobial resistance and hospital costs.

A recent survey among Dutch surgeons and residents demonstrated similar ambiguity concerning appendicitis with localized pus and gangrenous appendicitis: 61% and 38% of 80 respondents indicated they considered these types an indication for postoperative antibiotics, respectively [15]. Most commonly postoperative antibiotics were given for 3 days (58%) or 5 days (40%). Restricting postoperative antibiotics to less than 3 days was much less common (2,5%), compared to the 23% of respondents in this international survey that indicated this was the most common duration of prolonged prophylaxis. Two survey studies among pediatric surgeons in North America (published in 2003 and 2004) also addressed the postoperative management of complex (perforated) appendicitis [26, 39]. Both studies reported a highly variable duration of antibiotic therapy for perforated appendicitis. At that time, more than 90% of the pediatric surgeons extended intravenous prophylaxis beyond 3 postoperative days and added 4–10 days of oral antibiotics [26].

The lack of consistency in classification and management of appendicitis demonstrated in this survey was also addressed by Reid et al. [40]. They proposed a uniform intraoperative scoring system to more accurately define the type of appendicitis and predict the risk of recurrent abdominal infection. Likewise, a standardized definition of complex appendicitis is warranted to aid stratification of risk and guide postoperative antibiotic use [41]. According to the Surgical Infection Society, there are very little data on standardized approaches to prolonged prophylaxis for patients with complex appendicitis [10, 42]. It is suggested that standardized approaches to source control could improve outcomes. In pursuit of the shortest effective course, we recently started the APPIC trial, hypothesizing that 48 h of antibiotics is non-inferior to 5 days in terms of preventing infectious complications after surgery for complex appendicitis [43]. The present survey results imply that non-inferiority of the short 48 h course may significantly impact current practice.

One important limitation to this study is that it is unsure whether the respondents in this survey are a representative sample; therefore, the results may only be interpreted as an indication of variation in practice. To assess true variation in international current practice, one would have to perform an audit of appendicitis on a larger scale. This survey was built to encourage many responses in a short time frame. The questions were designed to minimize free-text responses, and the total number of questions was kept small. The focus was on different types of appendicitis as potential indications for prolonged prophylaxis and on the specifics of the antibiotic regimen. Other factors that may also influence postoperative management of complex appendicitis—such as preoperative and postoperative clinical characteristics or inflammatory biochemical results—were not addressed in this survey.

Despite these limitations, the results firmly suggest that there is considerable variability in the classification and postoperative management of patients with complex appendicitis. Future research should focus on identifying patients that benefit from prolonged antibiotic prophylaxis, determining the shortest effective course and standardizing the approach.

## Electronic supplementary material

Below is the link to the electronic supplementary material.

Supplementary material 1 (DOCX 19 kb)
