# Supplementary material for: Variation in Classification and Postoperative Management of Complex Appendicitis: A European Survey
Source: World J Surg. 2018 Sep 25;43(2):439–46. doi: 10.1007/s00268-018-4806-4 (PMC6329835; doi:10.1007/s00268-018-4806-4)
Supplement: Supplementary file 1 — Supplementary material 1 (DOCX 19 kb) [file 268_2018_4806_MOESM1_ESM.docx]

| TABLE S1. Work origin of respondents | | |
| --- | --- | --- |
| Country | **n** | **Region** |
| Belgium | 5 (4) | Western Europe |
| Bulgaria | 1 (1) | Eastern Europe |
| Croatia | 1 (1) | Eastern Europe |
| Denmark | 17 (12) | Northern Europe |
| Finland | 21 (15) | Northern Europe |
| Germany | 1 (1) | Western Europe |
| Greece | 1 (1) | Southern Europe |
| Ireland | 11 (8) | Western Europe |
| Italy | 4 (3) | Southern Europe |
| Lithuania | 13 (9) | Northern Europe |
| Norway | 15 (11) | Northern Europe |
| Poland | 2 (1) | Eastern Europe |
| Romania | 1 (1) | Eastern Europe |
| Spain | 1 (1) | Southern Europe |
| Sweden | 10 (7) | Northern Europe |
| Switzerland | 1 (1) | Western Europe |
| Turkey | 1 (1) | Eastern Europe |
| Ukraine | 1 (1) | Eastern Europe |
| United Kingdom | 30 (22) | Western Europe |
| Total | 137 |  |

SUPPLEMENTARY TABLES

| TABLE S2. Familiar with classification into simple and complex appendicitis? (n=137) | n (%) |
| --- | --- |
| Unfamiliar | 16 (16) |
| Familiar but don’t regularly use it | 26 (19) |
| Sometimes do, sometimes don’t use it | 27 (20) |
| (Almost) always use it | 68 (50) |

| TABLE S3. Preferred antibiotic agents | |
| --- | --- |
| Preferred for intravenous administration, n=128* | **n (%)** |
| Amoxicillin + clavulanate | 31 (22) |
| Other amoxicillin combination | 13 (10) |
| Cefuroxime + metronidazole | 38 (27) |
| Other cephalosporin + metronidazole | 13 (10) |
| Gentamicin + metronidazole | 8 (6) |
| Piperacillin + tazobactam | 17 (12) |
| Other piperacillin combination | 6 (5) |
| Other^‡^ | 3 (2) |
| Preferred for oral administration, n=127 | **N (%)** |
| Amoxicillin + clavulanate | 47 (37) |
| Other amoxicillin combination | 11 (9) |
| Cephalexin + metronidazole | 14 (11) |
| Other cephalosporin combination | 7 (6) |
| Ciprofloxacin + metronidazole | 31 (24) |
| Trimethoprim + sulfamethoxazole + metronidazole | 12 (9) |
| Other^§^ | 5 (4) |

^*^1 double answer in free text.

^‡^Other answers included: ciprofloxacin + metronidazole (2) and trimethoprim + sulfamethoxazole + metronidazole.

^§^Other answers included: clavulanate + metronidazole (1), levofloxacin + metronidazole (3) and tazobactam (1).
